# Effects of Statins on Renal Outcome in Chronic Kidney Disease Patients: A Systematic Review and Meta-Analysis

**DOI:** 10.1371/journal.pone.0132970

**Published:** 2015-07-07

**Authors:** Anawin Sanguankeo, Sikarin Upala, Wisit Cheungpasitporn, Patompong Ungprasert, Eric L. Knight

**Affiliations:** 1 Department of Internal Medicine, Columbia University College of Physicians and Surgeons, Bassett Medical Center, Cooperstown, New York, United States of America; 2 Department of Preventive and Social Medicine, Faculty of Medicine Siriraj Hospital, Mahidol University, Bangkok, Thailand; 3 Johns Hopkins University School of Public Health, Baltimore, Maryland, United States of America; 4 Division of Nephrology and Hypertension, Mayo Clinic, Rochester, Minnesota, United States of America; 5 Division of Rheumatology, Mayo Clinic, Rochester, Minnesota, United States of America; 6 Division of Nephrology, Columbia University College of Physicians and Surgeons, Bassett Medical Center, Cooperstown, New York, United States of America; Kaohsiung Medical University HospitalKaohsiung Medical University HospitalKaohsiung Medical University Hospital, TAIWAN

## Abstract

**Background:**

HMG CoA reductase inhibitors (statins) are known to prevent cardiovascular disease and improve lipid profiles. However, the effects of statins on renal outcomes, including decline in estimated glomerular filtration rate (eGFR) and proteinuria in patients with chronic kidney disease (CKD), are controversial. This meta-analysis evaluated the impact of statins on renal outcomes in patients with CKD.

**Materials and Methods:**

We comprehensively searched the databases of MEDLINE, EMBASE, and Cochrane Databases. The inclusion criteria were published RCT and cohort studies comparing statin therapy to placebo or active controls in patients with CKD (eGFR <60 ml/min/1.73 m^2^) not requiring dialysis. The primary outcome was the differences in the change of eGFR. We also examined change of protein concentration in urine as a secondary outcome. A meta-analysis comparing statin and its control groups and a subgroup analysis examining intensity of statin were performed.

**Results:**

From 142 full-text articles, 10 studies were included in the meta-analysis. Overall, there was a significant difference in rate of eGFR change per year favoring statin group (mean difference (MD) = 0.10 ml/min/1.73 m^2^, 95% CI: 0.09 to 0.12). In our subgroup analysis, those who received high-intensity statins had a significant difference in eGFR with a MD of 3.35 (95% CI: 0.91 to 5.79) ml/min/1.73 m^2^ compared to control. No significant change in eGFR was found with moderate- and low-intensity statin therapy. Compared with the control group, the statin group did not have a difference in reduction of proteinuria with MD in change of proteinuria of 0.19 gm/day (95% CI: -0.02 to 0.40).

**Conclusion:**

Overall, there was a difference in change of eGFR between the statin and control group. High-intensity statins were found to improve a decline in eGFR in population with CKD not requiring dialysis compared with control, but moderate- and low-intensity statins were not. Statins were not found to decrease proteinuria in patients with CKD.

## Introduction

Chronic kidney disease (CKD) is an important cause of death worldwide, affecting more than 10% of the population [1]. One of the risk factors for developing CKD and worsening renal outcomes is renovascular disease. One of the proposed mechanisms for progressive CKD in patients with renovascular disease is endothelial dysfunction, oxidative stress, and systemic inflammation of the glomerular capillary wall [2].

There is evidence that statins may improve renal function and lower proteinuria in many prospective cohort studies, randomized-control trials and meta-analyses [3–5]. This could be due to statin’s effects of decreased inflammation and improvement of endothelial function [6]. However, previous meta-analyses on the effect of statins on renal outcomes were not specifically done in CKD population [7]. One meta-analysis analyzed only the renal outcome at the end of treatment and did not examine change in renal function from baseline. Thus, the impact of statins on change in renal function in CKD patients is still unclear [8]. In addition, since the American College of Cardiology/American Heart Association (ACC/AHA) Guidelines [9] have emphasized different statin intensities in patients with different risk of atherosclerotic cardiovascular disease, we hypothesized that there is a dose-response relationship between statin intensities and renal outcome. Therefore, we conducted a systemic review with a meta-analysis of cohort studies and randomized-controlled trials to determine the effects of statins on change in renal function and protein excretion compared with controls in patients with CKD [10].

## Materials and Methods

This systematic review and meta-analysis was conducted and reported according to established guidelines [11,12] (S1 Appendix) and was registered in PROSPERO (registration number: CRD42014013047).

### Search Strategy

Two authors (AS and SU) independently searched published studies indexed in the Cochrane Central Register of Controlled Trials (CENTRAL) in The Cochrane Library, MEDLINE, and EMBASE from January 1995 to January 2015. There were no limitations on language or publication date. References of selected retrieved articles were also examined. Sample search terms were: “statin”, “Hydroxymethylglutaryl-CoA Reductase Inhibitors”, “hmg coa reductase inhibitor”, “chronic renal insufficiency”, “kidney failure”, “CKD”. We limited searches to human only. We did not use filter for study design or limit for adults. Search terms that were used are detailed in S2 Appendix.

### Inclusion and exclusion criteria

We included all published randomized clinical trials (RCTs), prospective cohort and retrospective cohort studies comparing statins with placebo or no statin therapy for at least 6 months in patients with chronic kidney disease. We included cohort studies to explore renal outcomes and potential side effects from statin use. We excluded reviews, case reports, letters, commentaries, abstracts, and unpublished articles.

We included participants aged 18 years or older who had CKD stages 3 to 4 (defined as eGFR 15–59 ml/min/1.73 m^2^) and had a baseline eGFR, creatinine clearance or protein concentration in urine. Participants who received dialysis, renal replacement therapy or renal transplantation were excluded from the analysis.

Primary outcome was change of eGFR or creatinine clearance from baseline. Secondary outcomes were change in urinary protein concentration, incidence of 50% reduction in eGFR, and incidence of ESRD.

### Data Extraction

Two authors (AS and SU) independently reviewed titles and abstracts of all citations that were identified. After all abstracts were reviewed, data comparisons between investigators were conducted to ensure completeness and reliability. The inclusion criteria were independently applied to all identified studies. Differing decisions were resolved by consensus.

Full-text versions of potentially relevant papers identified in the initial screening were retrieved. If multiple articles from the same study were found, only the article with the longest follow-up period was included. Data concerning study design, participant characteristics, interventions, and outcome measures were independently extracted. We contacted the authors of the primary reports to request any unpublished data. If the authors did not reply, we used the available data for our analyses.

### Assessment of Bias Risk

A subjective assessment of methodological quality for RCT’s was conducted by two authors (AS and SU) on the following items, in which each component was categorized as having high, low or unclear risk of bias: random sequence generation, allocation concealment, blinding of participants and personnel, blinding of outcome assessment, incomplete outcome data, and selective reporting. The quality of cohort studies was evaluated by the same authors using the Newcastle-Ottawa Scale (NOS) [13]. The NOS is a quality assessment tool for non-randomized study. It used a “star system” based on three major perspectives: the selection of the study groups (0–4 stars), the comparability of the groups by controlling for first and second most relevant factors (0–2 stars), and the ascertainment of outcome of interest (0–3 stars). A total score of 3 or less was considered poor, 4–6 was considered moderate, and 7–9 was deemed high quality. We excluded studies from our meta-analysis if they had poor quality. Discrepant opinions between authors were resolved by consensus.

### Statistical Analysis

We performed meta-analysis of RCT and cohort studies separately. We reported the pooled mean difference (MD) of a total change in eGFR, proteinuria, rate of change in eGFR per year between statin group and controls. Pooled risk ratio (RR) of 50% reduction of eGFR and incidence of ERSD were also reported. For the purpose of our meta-analysis, creatinine clearance was considered to be equivalent to eGFR. The extracted studies were excluded from the analysis if they did not present an outcome in each of the intervention groups or did not have enough information required for continuous data comparison.

We also performed a subgroup analysis of statin intensity, which was characterized as “high-intensity”, “moderate-intensity”, and “low-intensity” statin therapy, using definitions from the recent ACC/AHA guidelines (9). High intensity statins lower LDL-C by approximately ≥ 50%, include atorvastatin 40–80 mg and rosuvastatin 20-(40) mg. Moderate-intensity statins lower LDL-C by approximately 30% to <50%, include atorvastatin 10-(20) mg, rosuvastatin (5)-10 mg, simvastatin 20–40 mg, pravastatin 40-(80) mg, lovastatin 40 mg, fluvastatin XL 80 mg, fluvastatin 40 mg bid, pitavastatin 2–4 mg. Low-intensity statins lower LDL-C by approximately <30%, include fluvastatin 20–40 mg, lovastatin 20 mg, simvastatin 10 mg, pitavastatin 1 mg, pravastatin 10–20 mg. Statin and doses that are approved by the U.S. FDA but were not tested in the RCTs reviewed in the guidelines are listed in parentheses.

The heterogeneity of effect size estimates across these studies was quantified using the I^2^ statistic and Q statistic. For Q statistic, substantial heterogeneity was defined as P<0.1. The I^2^ statistic ranges in value from 0 to 100% (I^2^<25%, low heterogeneity; I^2^ = 25%–50%, moderate heterogeneity; and I^2^>50%, substantial heterogeneity). A sensitivity analysis was performed to assess the influence of the individual studies on the overall results by omitting one study at a time. Meta-regression was performed to find source of heterogeneity and to assess association between baseline LDL-C or percent reduction in LDL-C in the statin group and difference in eGFR change. Possible publication bias was assessed using funnel plot and Egger’s regression test [14] (P<0.05 was considered significant). All data analyses were performed using the Comprehensive Meta-Analysis 3.3 software from the Biostat, Inc.

## Results

### Description of Included Studies

**Fig 1:**
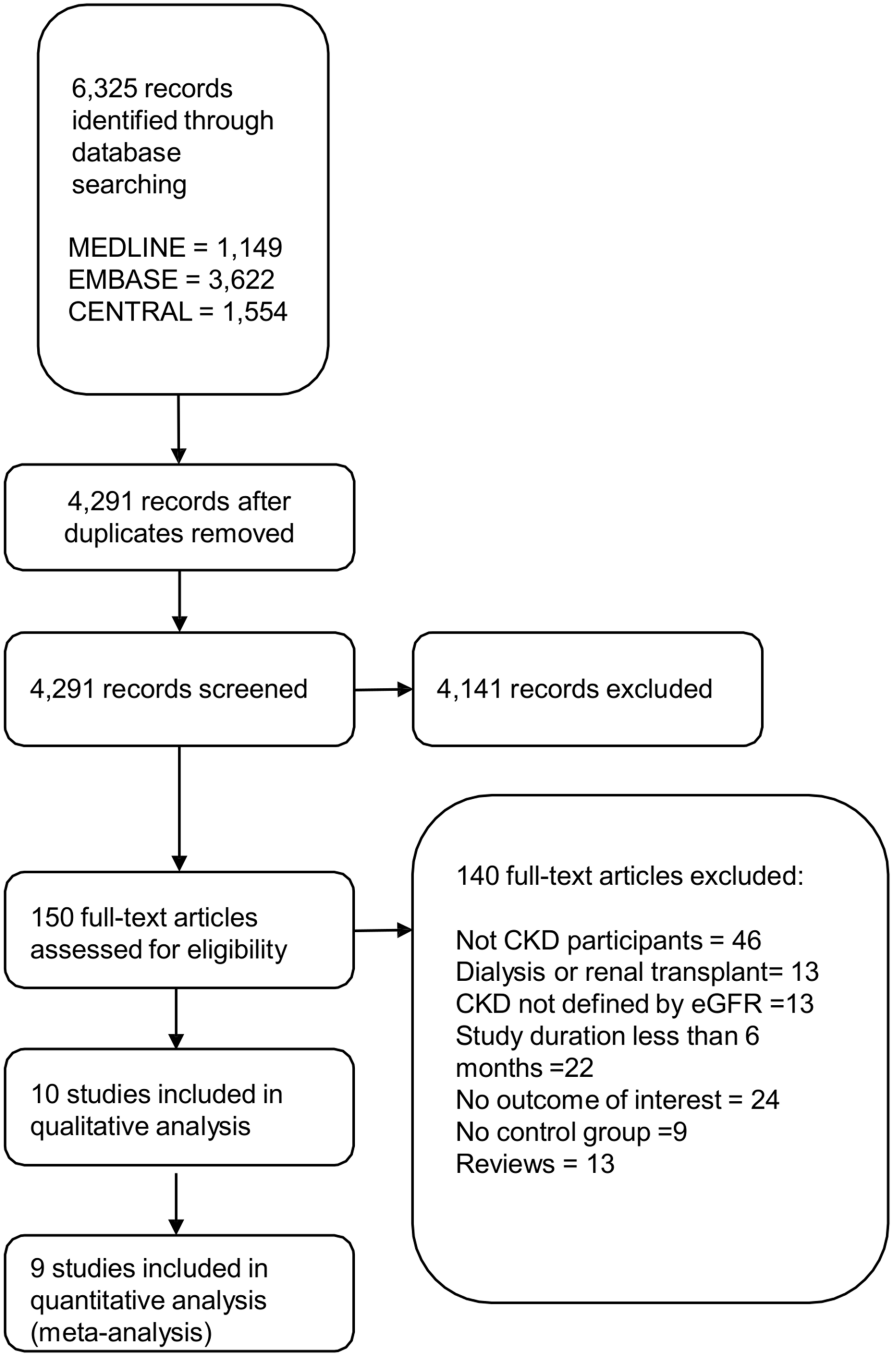
Results of Information Search.

The initial search yielded 4,291 articles (Fig 1); 4,141 articles were excluded because they were not RCTs or observational studies (1,866 articles), did not involve CKD participants (1,426 articles), included dialysis patients or did not have primary outcome (849 articles). A total of 150 articles underwent full-length review. Data were extracted from ten studies involving total of 18,126 CKD participants for qualitative analysis [15–24]. The included studies varied in sample size (38 to 6,245), type and dose of statins used (Fluvastatin 20 mg, Pravastatin 40 mg, Atorvastatin 40–80 mg, Lovastatin 40 mg, and Rosuvastatin 2.5 mg), and duration of treatment (12–64 months). Among the ten included studies, nine were RCT’s, and one was a prospective cohort study. Sample size of the included studies ranged from thirty-eight to 6,245. Low, moderate and high-intensity statins were all used in the included studies. The study duration ranged from twelve to sixty-three months. LDL-C reduction from baseline ranged from 7.6% to 40.4%. The characteristics of the ten extracted studies included in this review are outlined in Table 1.

**Table 1:** Characteristics of included studies.

Study	Design	Follow-up period (months)	Characteristics			Participants (n)		Comorbidities	Intervention	Statin intensity	Control	Baseline eGFR (ml/min/1.73 m^2^)		Baseline urinary protein excretion (g/day)		Baseline LDL-C (mg/dL)	LDL-C reduction (Statin group)	Side effects*
			Age	Female (%)	Race	Treatment	Control					Statin	Control	Statin	Control			
Yasuda 2004 [15]	Prospective, open-label RCT	12	57	53.8	Japanese (100%)	39	41	DM (100%)	Fluvastatin 20 mg plus diet	Low	Dietary	59 ± 31.2	60 ± 25.6	0.8 ± 1.2	0.7 ± 0.6	170.1	25%	None
Tonelli 2003 [16]	Randomized double- blind placebo controlled trial	36	63	24	White (97.7%)	345	345	CHD (100%) DM (15.4%)	Pravastatin 40 mg	Moderate	Placebo	53.2 ±6.0	52.5 ±6.5	-	-	139.5	32%	None
Tonelli 2005 [17]	Randomized double- blind controlled trial	60	63	23.6	Black (0.5%)	1702	1700	Hypercholesterolemia (100%), CHD (100%), DM (10.6%)	Pravastatin 40 mg	Moderate	Placebo	52.7 ± 6.1	52.7 ± 5.9	-	-	154.0	-	Non-dermatologic malignancy
Bianchi 2003 [18]	Prospective, controlled open-label RCT	12	55.6	32.1	N/A	28	28	HTN (48.2%)	Atorvastatin 40 mg	High	No atorvastatin	50.8 ± 9.5	50.0 ± 10.1	2.2 ± 0.5	2.1 ± 0.5	203.0	40.4%	N/A
Kendrick 2009 [19]	Randomized double- blind placebo controlled trial	64	62	21.3	African-American (1.3%)	145	159	HTN (35.2%), DM (1.6%)	Lovastatin maximum dose 40 mg	Moderate	Placebo	53 ± 6	53 ± 6	-	-	151.0	27%	None
Rahman 2008 [20]	Prospective randomized clinical trial	58	70.7	54.4	White non-Hispanic (50.7), Black non-Hispanic (28.6), White Hispanic (13.9), Black Hispanic (1.4), Other (5.3)	779	778	HTN (100%) DM (30.8%)	Pravastatin 40 mg	Moderate	Usual care	50.8 ± 8.2	50.6 ± 8.4	-	-	146.5	30.2%	None
Koren 2009 [21]	Prospective, open-label RCT	54	65.2	23.1	White (88.1%), African-American (8.5%)	286	293	CHD (100%), DM (28%)	Atorvastatin 80 mg	High	Usual care	51.3 ± 7.8	51.1 ± 8.5	-	-	148.0	34.5%	N/A
Sawara 2008 [22]	Prospective, open-label RCT	12	67	47.4	Japanese (100%)	22	16	CHD (34.2%)	Rosuvastatin 2.5 mg	Moderate	No lipid lowering drugs	50.7 ± 18.7	57.3 ± 16.2	0.17 ± 0.29	0.12 ± 0.3	130.3	24.3%	N/A
Natsuaki 2012^a^ [23]	Prospective cohort	31	71.4	20.8%	Japanese (100%)	2135	2432	CHD (100), HTN (92.6%), DM (54.4%)	statins	N/A	No statin	49.1 ± 7.7	48.5 ± 8.0	-	-	119.0	15.9%	N/A
Natsuaki 2012^b^ [23]	Prospective cohort	31	72	39.8%	Japanese (100%)	229	379	CHD (100), HTN (92.6%), DM (54.4%)	statins	N/A	No statin	22.0 ± 6.2	20.8 ± 6.7	-	-	114.0	7.6%	N/A
Haynes 2014 [24]	Randomized placebo controlled trial	48	63	37.8%	White (71.2%), Black (1.9%), Asian (24.5%), Other (1.9%)	3116	3129	DM (22.8%)	Simvastatin 20 mg plus exetimibe 10 mg	Moderate	Placebo	26.6 ± 12.9	26.6 ± 13.1	217 (44–787)	196 (43–746)	111.0	31.0%	N/A

Values are presented as mean ± SD or median (interquartile range).

^a^ Mild CKD (eGFR ≥30– <60 ml/min/1.73 m^2^)

^b^ Severe CKD (eGFR <30 ml/min/1.73 m^2^)

* Significant side effects (p<0.05) in the statin group compared with the control group.

Abbreviation: CHD = coronary heart disease, DM = diabetes mellitus, eGFR = estimated glomerular filtration rate, HTN = hypertension, LDL = Low-density lipoprotein, RCT = Randomized controlled trial.

Of these, six studies were included in the meta-analysis for difference in eGFR change [15,18,20,21,22] and rate of eGFR change per year [16,17,19,20,24]. Three studies were included in the analysis of difference in proteinuria [15,22,24]. Two studies were included in analysis comparing 50% reduction of eGFR and incidence of ESRD [20,24]

### Risk of Bias of Included Studies

All RCT and non-randomized prospective controlled studies were assessed for risk of bias (summarized in S1 Fig). About one-fourth of the studies had selection bias because of non-randomization or no allocation concealment. In addition, only one-third of studies were double-blinded and had blinding of outcome assessment. The quality of one prospective cohort study [23] was evaluated by NOS. It received a total of 8 stars (4 stars for selection, 2 stars for comparability, and 2 stars for ascertainment of outcome).

### Meta-analysis Results

**Fig 2:**
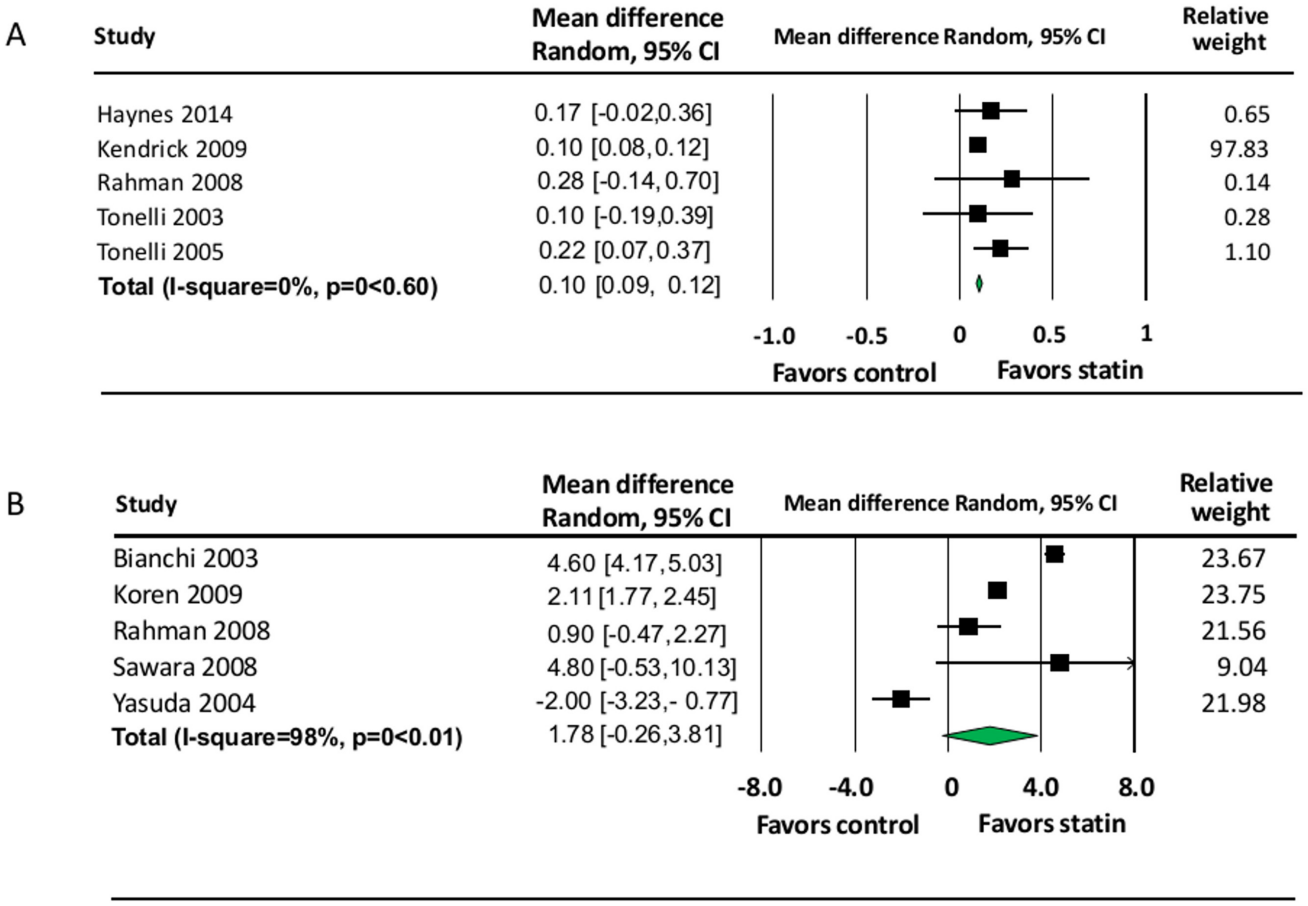
Comparison of eGFR Change Between Statin and Control Groups. A) Rate of eGFR Change Per Year (ml/min/1.73 m^2^), B) Total Change in eGFR.

We performed this meta-analysis to investigate the effects of statins versus placebo or control on eGFR and proteinuria using a random effects model. Since there is only one prospective cohort study [23], only RCTs were included in the meta-analyses. There was significant difference in the change in the average end of treatment eGFR for rate of eGFR change per year, but no difference the total change of eGFR (Fig 2A and 2B **)**. The MD of rate of eGFR change per year was 0.10 ml/min/1.73 m^2^ (95% confidence interval [CI]: 0.09 to 0.12). The statistical between-study heterogeneity was absence (I^2^ = 0%, p-value = 0.60). The MD of total change in eGFR was 1.78 ml/min/1.73 m^2^ (95% CI: -0.26 to 3.81). The statistical between-study heterogeneity was statistically significant with an I^2^ of 98% (p-value<0.01).

A meta-analysis of studies examining change in proteinuria was also performed **(** S2 Fig **)**. There was no significant change in urinary protein excretion with a MD of 0.19 gm/day (95% CI: -0.02 to 0.40). There was significant heterogeneity between the included studies in this meta-analysis (I² = 90%).

Only two studies were included in the analysis of comparison in incidence of ESRD and 50% reduction of eGFR or ESRD between statin and control groups. There were no significant association between statin group and control with ESRD (RR = 0.97, 95% CI: 0.90 to 1.06), and 50% reduction of eGFR (RR = 0.93, 95% CI: 0.86 to 1.01) (S3A and S3B Fig).

**Fig 3:**
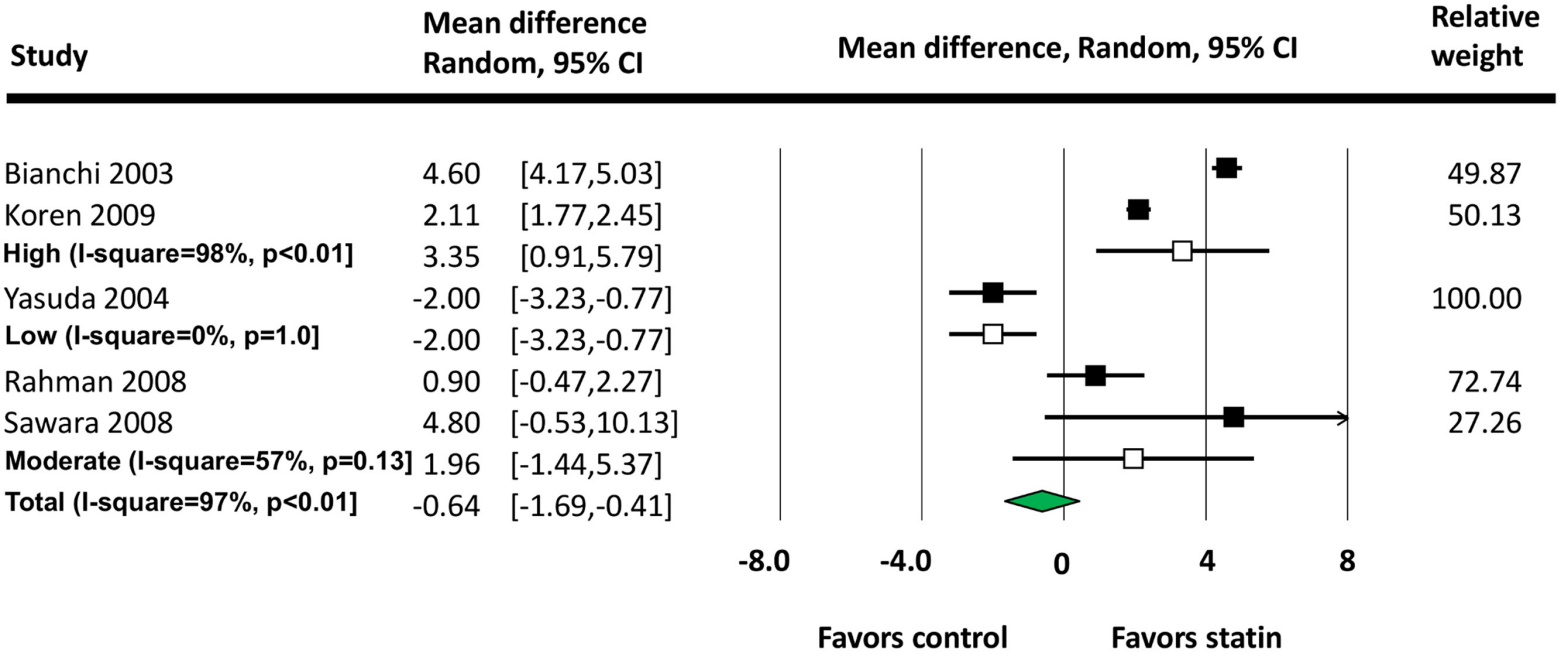
Subgroup Analysis of Total Change in eGFR (ml/min/1.73 m^2^) in High, Moderate and Low Intensities Statin Groups.

The results of our subgroup analysis of statin intensity is shown (Fig 3). There was a significant increase in eGFR in studies of high-intensity statin (LDL-C reduction = 34%-40%) compared with control: the MD of total change in eGFR was 3.35 ml/min/1.73 m^2^ (95% CI: 0.91 to 5.79). No statistically significant difference of change in eGFR was found with moderate- (LDL-C reduction = 24%-32%) and low- (LDL-C reduction = 25%) intensity statin therapy. There were insufficient data to investigate effects of statins intensities on proteinuria.

### Sensitivity Analysis

To assess the stability of the results of the meta-analysis of comparison in total change of eGFR and rate of eGFR change, sensitivity analyses were conducted by excluding one study at a time. None of the results was significantly altered, indicating that our results were robust.

### Meta-regression

We performed meta-regression analysis using random-effects model to explore the effects of covariates (percent reduction in LDL and baseline LDL) on differences in total change of eGFR. Percent reduction in LDL-C was significant with a coefficient of -9.9 (95% CI: -18.2 to -1.5), p = 0.02, and R^2^ = 60% (S4 Fig). In the model that explored effects of baseline LDL-C on change in eGFR, it was a significant predictor with a coefficient of 0.04 (95% CI: 0.03 to 0.05, p<0.01 and R^2^ = 98%.

### Publication Bias

To investigate potential publication bias, we examined the contour-enhanced funnel plot of the included studies in assessing total change in eGFR. Vertical axis represents study size (standard error) while horizontal axis represents effect size (log odds ratio). From this plot, bias is not present because there is symmetrical distribution of studies on both sides of the mean. The results of Egger’s test suggest that no evidence of publication bias was observed (P = 0.58). The funnel plot for assessing change in proteinuria was not performed due to too few studies.

## Discussion

### Summary of the Main Results

Overall, our systematic review and meta-analysis in CKD participants found that high-intensity statin therapy [18,21] had a significantly less reduction in eGFR compared to control. The total difference in eGFR was 3.35 ml/min/1.73 m^2^ in the high-intensity statin group compared with control. However, we found no difference with moderate- and low-intensity statin therapy. In addition, we did not find that statins lowered proteinuria compared to the control group.

### Agreements and Disagreements with Other Studies or Reviews

Our findings are consistent with other meta-analysis in CKD participants not requiring dialysis [25,26], which found marginal benefits of statins on change in GFR compared with controls. However, those meta-analyses found a greater reduction of proteinuria in the statin group. We believed that the difference in our findings could be due to a difference in inclusion and exclusion criteria of eligible studies.

Previous meta-analyses of studies that reported renal function found that the effect of statins on eGFR may be dose-related in a general population [7]. However, our study is unique in that our findings were specifically done in CKD participants with baseline eGFR less than 60 ml/min/1.73 m^2^. It is possible that CKD patients had greater baseline renovascular disease and therefore required the greater effect of high intensity statins to provide a benefit on renal vasculature and to reduce greater oxidative stress. In addition, we demonstrated that LDL-C reduction had a significant association with change in eGFR. There is an increased change of eGFR with a greater reduction of LDL-C. This analysis helps to confirm a higher change of eGFR in high-intensity statins that had greater reduction of LDL-C compared with moderate- and low-intensity statins. Underlying mechanism of increased change of eGFR might be related to a higher reduction of oxidized LDL particles that normally cause renal and vascular damage. We also found an increased change of eGFR in higher baseline LDL-C. Higher baseline LDL-C may cause more renal injury than lower LDL-C. More severe renal injury may benefits from statin therapy more than lower injury [27,28].

Our findings are also unique in that we studied the effects of statin on hard end-points such as ESRD or 50% reduction of eGFR. We did not find a significant association of statins and these outcome. Our findings might be useful for clinicians to use high-intensity statin therapy to slow deterioration in renal function in CKD patients. However, there are no clear benefits of statin therapy on proteinuria. This might be because renal injury from lipotoxicity is independent of proteinuria [29].

### Strengths and Limitations

Strengths of our study include only enrolling CKD participants with a baseline eGFR less than 60 ml/min/1.73 m^2^ and a comprehensive MEDLINE, EMBASE and Cochrane Databases database search. Also, we included only studies with a treatment duration of at least 6 months, which allowed us to see the longer term effects of statins on renal function. In addition, we analyzed the difference in the change of renal function, and not only the end of treatment outcome as in a previous meta-analysis examining this topic [26]. Furthermore, we did a subgroup analysis on statin intensity, which can keep answer whether statins may have a dose-response effect in CKD patients.

There are several limitations in our meta-analysis, and thus our results should be interpreted with caution. First, we included a relatively small number of studies. This is because we separately analyzed studies with the outcome of total change in eGFR and rate of eGFR change per year as they used different units and we did not have enough information to change them into the same unit.

Second, there is high heterogeneity between studies in the meta-analysis of statins effect on total change of eGFR. Potential sources of heterogeneity assessed by meta-regression were study designs, baseline LDL-C, and LDL-C reduction. We found that there was an increased in total change of eGFR with a higher baseline LDL-C or with an increased in reduction of LDL-C. This finding supports our main results of a significant effects of high-intensity statin.

## Conclusions

Our results add evidence that statin use in CKD patients may delay progression of kidney disease, and our results suggest that statins may have a dose-related effect on kidney function as only high-intensity statin significantly improved renal function assessed by estimated GFR. However, these results should be interpreted with caution. Further RCT’s using different statin intensities in CKD patients not requiring dialysis with a longer duration of study are needed.

## Supporting Information

S1 Appendix PRISMA checklist. (DOC)

S2 Appendix Search Strategy. (DOCX)

S1 Fig Risk of Bias of included studies. Review authors' judgments about each risk of bias. Item presented as percentages across all RCT and Non-Randomized trials. Positive signs represent low risk of bias. Negative signs represent high risk of bias. Blank spaces represent unclear risk.
(TIF)

S2 Fig Comparison of Change in Urinary Protein Excretion Between Statin and Control Groups. (TIF)

S3 Fig Comparison of Risk of A) 50% Reduction in eGFR and B) ESRD Between Statin and Control Groups. (TIF)

S4 Fig Meta-Regression Scatter Plot of Studies Comparing Total Change in eGFR Using Percent Reduction in LDL-C. Circles represent each included study.
(TIF)
