# Supplementary material for: Effects of Statins on Renal Outcome in Chronic Kidney Disease Patients: A Systematic Review and Meta-Analysis
Source: PLoS One. 2015 Jul 7;10(7):e0132970. doi: 10.1371/journal.pone.0132970 (PMC4495033; doi:10.1371/journal.pone.0132970)
Supplement: S2 Appendix — (DOCX) [file pone.0132970.s002.docx]

**Search strategies**

MEDLINE

1. Hydroxymethylglutaryl-CoA Reductase Inhibitors.mp. or exp Hydroxymethylglutaryl-CoA Reductase Inhibitors/

2. simvastatin.mp. or exp Simvastatin/

3. atorvastatin.mp.

4. fluvastatin.mp.

5. pravastatin.mp. or exp Pravastatin/

6. pitavastatin.mp.

7. lovastatin.mp. or exp Lovastatin/

8. hmg coa reductase inhibitor*.mp.

9. hydroxymethylglutaryl coenzyme a reductase inhibitor*.mp.

10. hmg co a reductase inhibitor*.mp.

11. mevinolin.mp.

12. pravachol.mp.

13. lipitor.mp.

14. zocor.mp.

15. mevacor.mp.

16. lescol.mp.

17. statin*.mp.

18. 1 or 2 or 3 or 4 or 5 or 6 or 7 or 8 or 9 or 10 or 11 or 12 or 13 or 14 or 15 or 16 or 17

19. renal insufficiency, chronic.mp. or exp Renal Insufficiency, Chronic/

20. exp Kidney Failure, Chronic/ or kidney failure, chronic.mp.

21. uremia.mp. or exp Uremia/

22. CKD.mp.

23. CRD.mp.

24. CRF.mp. [mp=title, abstract, original title, name of substance word, subject heading word, keyword heading word, protocol supplementary concept word, rare disease supplementary concept word, unique identifier]

25. CKF.mp. [mp=title, abstract, original title, name of substance word, subject heading word, keyword heading word, protocol supplementary concept word, rare disease supplementary concept word, unique identifier]

26. chronic renal.mp.

27. chronic kidney.mp.

28. uremi*.mp.

29. 19 or 20 or 21 or 22 or 23 or 24 or 25 or 26 or 27 or 28

30. 18 and 29

EMBASE

(('hydroxymethylglutaryl coa reductase inhibitors'/exp or 'hydroxymethylglutaryl coa reductase inhibitors' and [embase]/lim) or ('simvastatin'/exp or 'simvastatin' and [embase]/lim) or ('atorvastatin'/exp or 'atorvastatin' and [embase]/lim) or ('fluvastatin'/exp or 'fluvastatin' and [embase]/lim) or ('pravastatin'/exp or 'pravastatin' and [embase]/lim) or ('pitavastatin'/exp or 'pitavastatin' and [embase]/lim) or ('lovastatin'/exp or 'lovastatin' and [embase]/lim) or ('hmg coa reductase inhibitor' and [embase]/lim) or ('hydroxymethylglutaryl coenzyme a reductase inhibitor' and [embase]/lim) or ('hmg co a reductase inhibitor' and [embase]/lim) or ('mevinolin'/exp or 'mevinolin' and [embase]/lim) or (pravachol and [embase]/lim) or (lipitor and [embase]/lim) or (zocor and [embase]/lim) or (mevacor and [embase]/lim) or (lescol and [embase]/lim) or (statin* and [embase]/lim)) and (('renal insufficiency chronic'/exp or 'renal insufficiency chronic' and [embase]/lim) or ('kidney failure chronic'/exp or 'kidney failure chronic' and [embase]/lim) or ('uremia'/exp or 'uremia' and [embase]/lim) or (ckd and [embase]/lim) or (crd and [embase]/lim) or (crf and [embase]/lim) or (ckf and [embase]/lim) or ('chronic renal' and [embase]/lim) or ('chronic kidney' and [embase]/lim) or (uremi* and [embase]/lim))

CENTRAL

#1 MeSH descriptor: [Hydroxymethylglutaryl-CoA Reductase Inhibitors] explode all trees 2806

#2 MeSH descriptor: [Simvastatin] explode all trees 1212

#3 MeSH descriptor: [Pravastatin] explode all trees 869

#4 MeSH descriptor: [Lovastatin] explode all trees 1522

#5 atorvastatin 2956

#6 fluvastatin 628

#7 pitavastatin 168

#8 hydroxymethylglutaryl-coa reductase inhibitor* 2916

#9 hmg coa reductase inhibitor* 757

#10 hydroxymethylglutaryl coenzyme a reductase inhibitor* 1224

#11 hmg co a reductase inhibitor* 108

#12 simvastatin 2459

#13 lovastatin 926

#14 Pravastatin 1622

#15 mevinolin 115

#16 pravachol 21

#17 lipitor 42

#18 zocor 53

#19 mevacor 24

#20 lescol 68

#21 statin* 5228

#22 #1 or #2 or #3 or #4 or #5 or #6 or #7 or #8 or #9 or #10 or #11 or #12 or #13 or #14 or #15 or #16 or #17 or #18 or #19 or #20 or #21 10053

#23 MeSH descriptor: [Renal Insufficiency, Chronic] explode all trees 3798

#24 MeSH descriptor: [Kidney Failure, Chronic] explode all trees 3427

#25 MeSH descriptor: [Renal Dialysis] explode all trees 4353

#26 MeSH descriptor: [Uremia] explode all trees 406

#27 CKD 1152

#28 CRD 66040

#29 CRF 630

#30 CKF 48

#31 chronic renal 9389

#32 chronic kidney 7937

#33 uremi* 977

#34 #23 or #24 or #25 or #26 or #27 or #28 or #29 or #30 or #31 or #32 or #33 78052

#35 #22 and #34 1554
