# Supplementary figures and images for: Effects of Statins on Renal Outcome in Chronic Kidney Disease Patients: A Systematic Review and Meta-Analysis
Source: PLoS One. 2015 Jul 7;10(7):e0132970. doi: 10.1371/journal.pone.0132970 (PMC4495033; doi:10.1371/journal.pone.0132970)

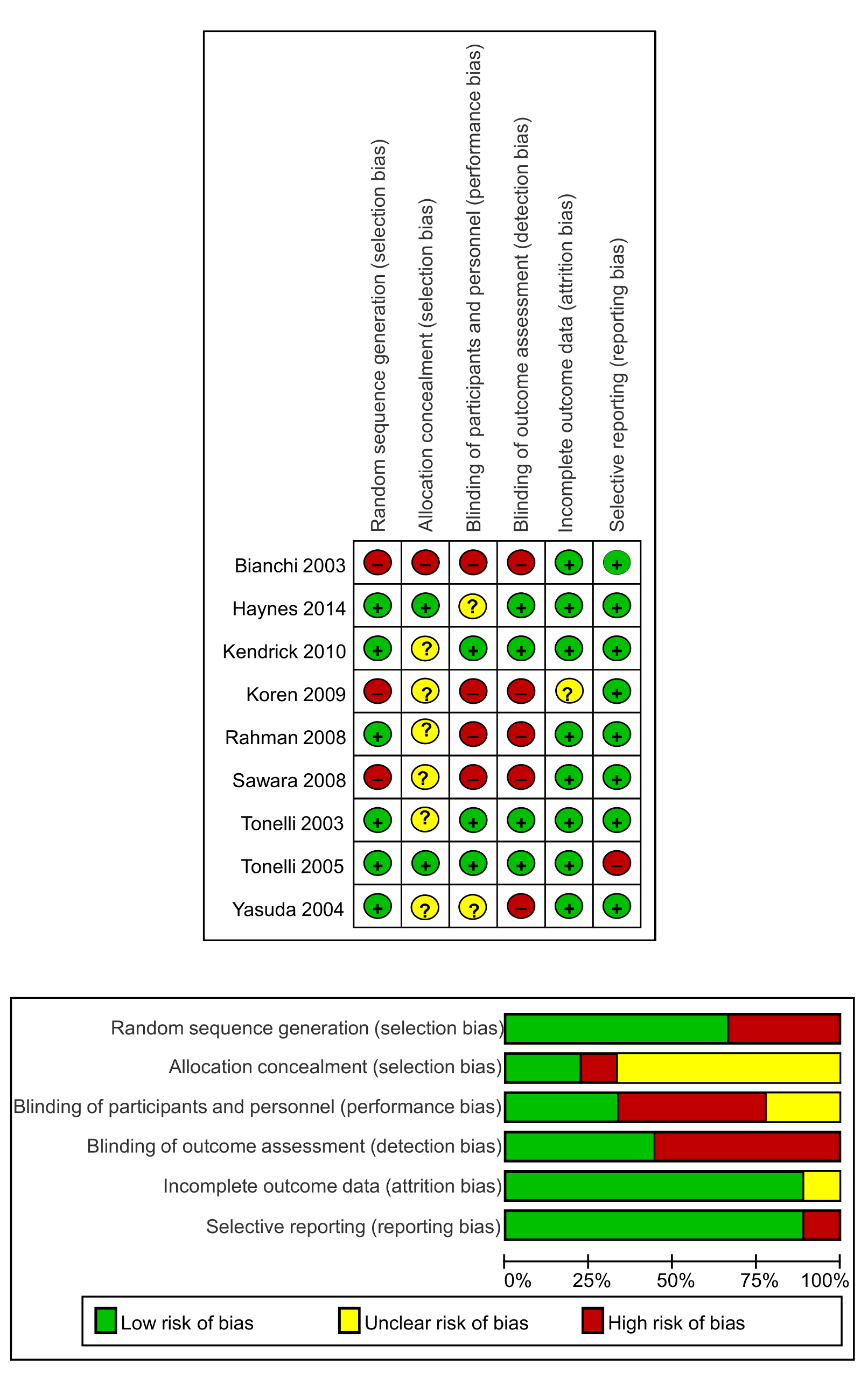

Supplement: S1 Fig — Review authors' judgments about each risk of bias. Item presented as percentages across all RCT and Non-Randomized trials. Positive signs represent low risk of bias. Negative signs represent high risk of bias. Blank spaces represent unclear risk. (TIF) [file pone.0132970.s003.tif]

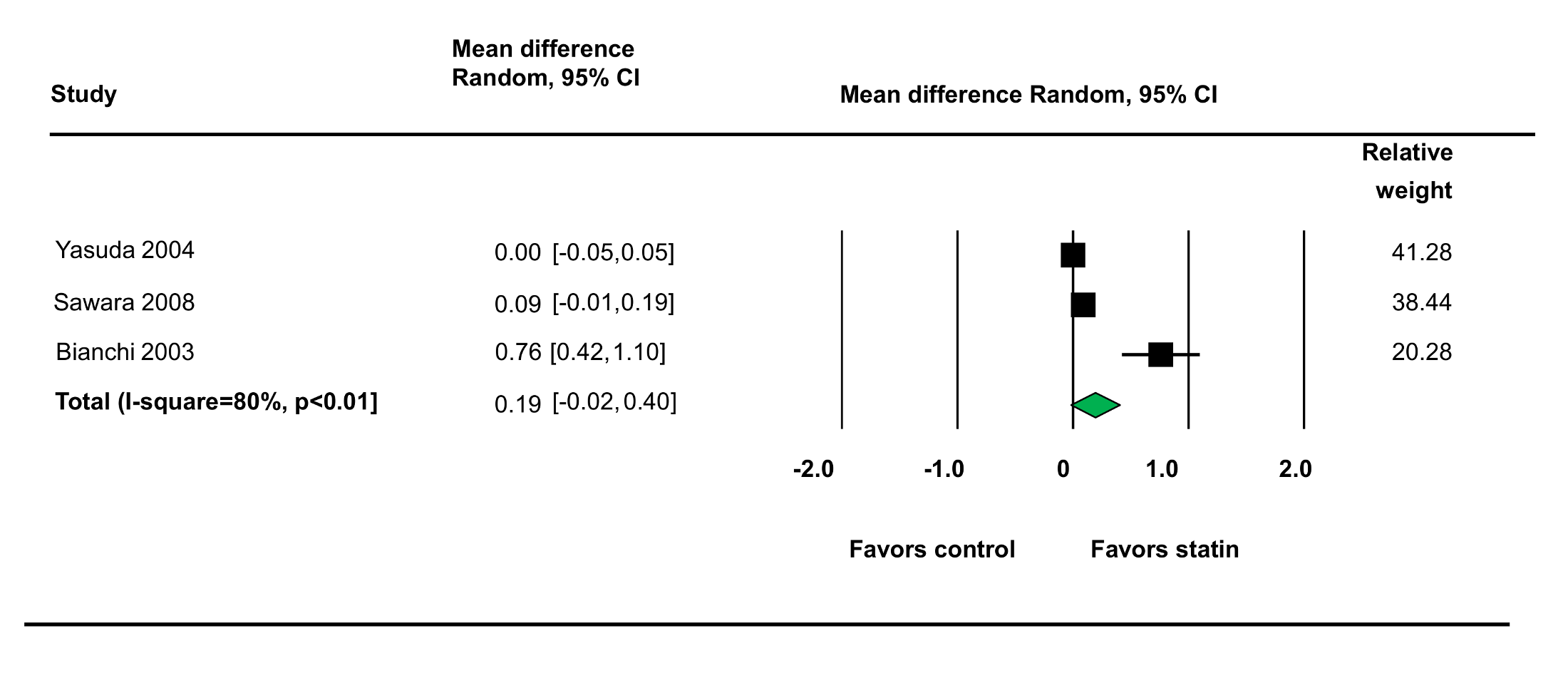

Supplement: S2 Fig — (TIF) [file pone.0132970.s004.tif]

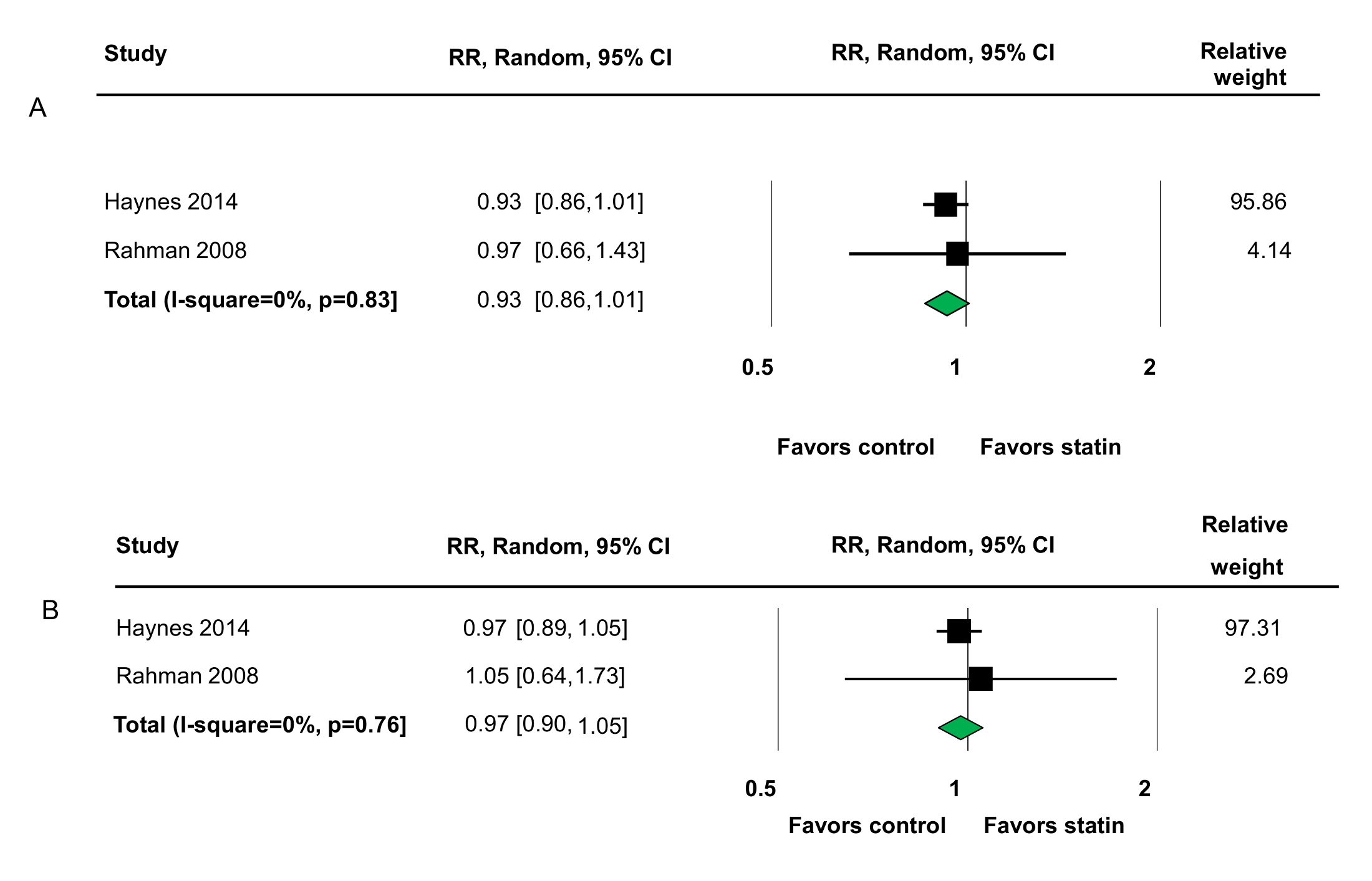

Supplement: S3 Fig — (TIF) [file pone.0132970.s005.tif]

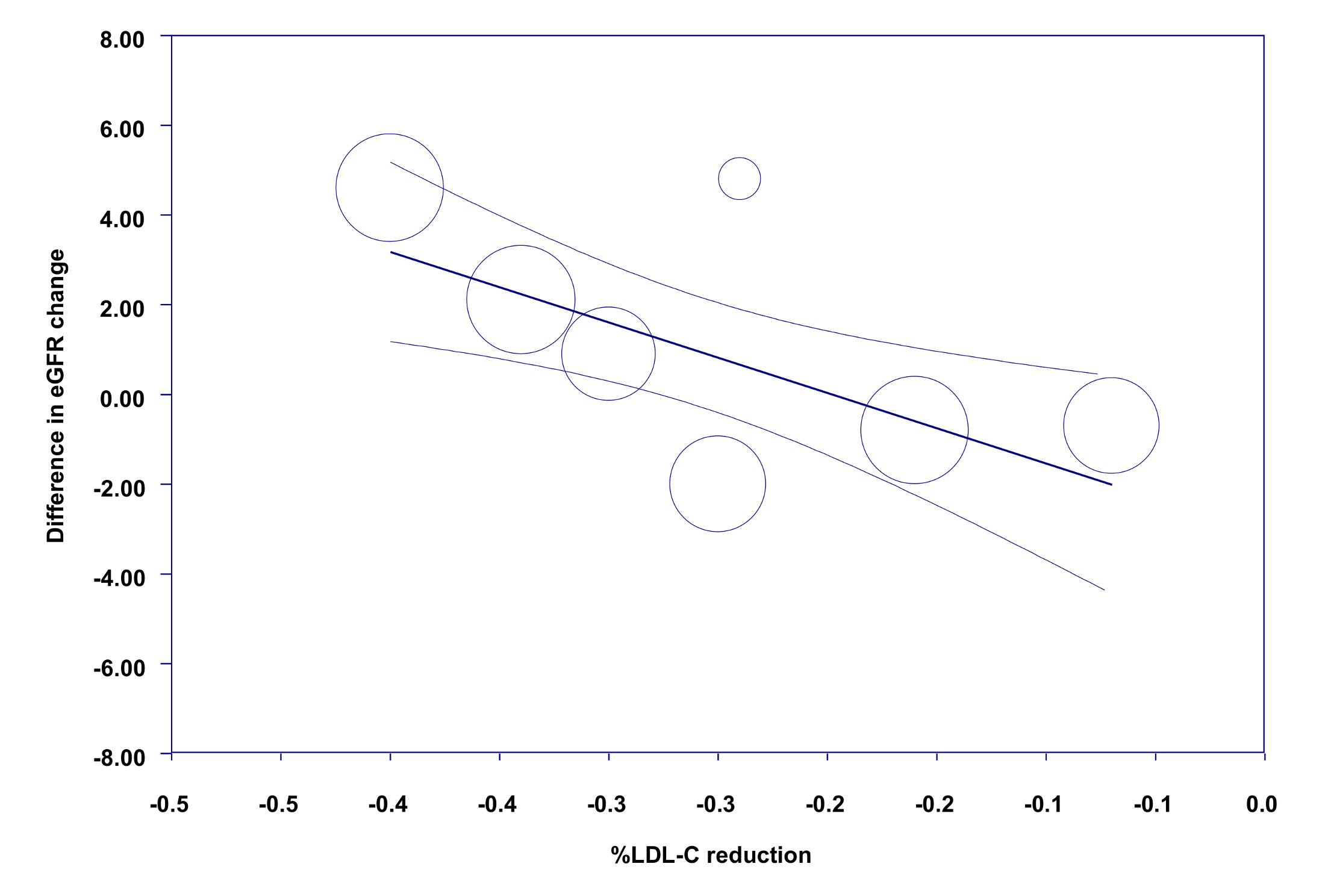

Supplement: S4 Fig — Circles represent each included study. (TIF) [file pone.0132970.s006.tif]
